# The Precise Diagnosis of Wolfram Syndrome Type 1 Based on Next-Generation Sequencing

**DOI:** 10.3389/fgene.2019.01217

**Published:** 2019-11-26

**Authors:** Dan-Dan Wang, Fang-Yuan Hu, Feng-Juan Gao, Sheng-Hai Zhang, Ping Xu, Guo-Hong Tian, Ji-Hong Wu

**Affiliations:** ^1^Eye Institute, Eye and ENT Hospital, College of Medicine, Fudan University, Shanghai, China; ^2^Shanghai Key Laboratory of Visual Impairment and Restoration, Science and Technology Commission of Shanghai Municipality, Shanghai, China; ^3^Key Laboratory of Myopia, Ministry of Health, Shanghai, China

**Keywords:** wolfram syndrome, WFS1, optic atrophy, diabetes mellitus, next-generation sequencing, precise diagnosis

## Abstract

**Purpose:** To explore a method for the early, rapid and accurate diagnosis of Wolfram syndrome 1 (WS1) and further enrich the spectrum of *WFS1* mutations in the Chinese population.

**Methods:** We analyzed 279 patients with unexplained optic atrophy using next-generation sequencing. All patients underwent detailed clinical evaluations. Furthermore, Sanger sequencing and cosegregation analyses were performed within families.

**Results:** Five patients with WS1 were identified in four unrelated families, and their clinical features were reviewed in detail. Seven variants of *WFS1* were detected, including three reported variants (p.G674R, p.Tyr508Cysfs*34, and p.G702D) and four novel variants (p.W540G, p.K634*, p.F770C, and p.Q584P). Furthermore, the variant p.G674R was recurrent.

**Conclusion:** Considering that WS1 is a rare progressive neurodegenerative disease, early diagnosis is beneficial to the systematic evaluation, monitoring and management of complications to improve patient quality of life and delay the progression of the disease. In the future, precise diagnosis on the basis of clinical manifestation and genetic testing will become the gold standard for the diagnosis of hereditary eye diseases and syndromes. Finally, our results further increase the spectrum of *WFS1* mutations by adding four novel variants to the limited data available in the Chinese population.

## Introduction

Wolfram syndrome 1 (WS1, MIM# 222300) is a rare inherited disease, characterized by diabetes insipidus), diabetes mellitus (DM), optic atrophy (OA), and deafness, and is therefore also known as DIDMOAD. It was first described in 1938 (Wolfram and Wagener, 1938). Other findings include cataracts, pigmentary retinopathy, hydronephrosis, hydroureter, neurogenic bladder, ataxia, and psychiatric disorders. Patients may also present with cardiomyopathy, hypothyroidism, and hypogonadism. The estimated prevalence ranges from 1:68,000 to 1:770,000 (Fraser and Gunn 1977; Barrett et al., 1995; Medlej et al., 2004; Matsunaga et al., 2014). The age at death varies from 25 to 49 years (median age is 30 years) and is usually due to brain stem atrophy resulting in respiratory failure (Barrett et al., 1995). There is currently no effective treatment. The clinical diagnostic criteria for WS1 are juvenile-onset DM and OA. It can be easily misdiagnosed as type 1 DM before other symptoms, such as OA, present. It is crucial to combine genetic diagnosis with clinical manifestations to obtain an early diagnosis and confirmation of WS1.

WS1 is an autosomal recessive condition, caused by mutations in the *WFS1* gene [Online Mendelian Inheritance in Man (OMIM) 606201]. The *WFS1* gene is mapped to human chromosome 4p16.1 (Strom et al., 1998) and is composed of eight exons, spanning 33.4 kb of genomic DNA. Exon 8 is the largest exon, and exons 2–7 are small coding exons, and exon 1 is noncoding (Inoue et al., 1998). *WFS1* encodes wolframin, a transmembrane protein that localizes primarily in endoplasmic reticulum (ER) membranes and is ubiquitously expressed at the highest levels in the brain, pancreas, heart, and insulinoma beta-cell lines (Hofmann et al., 2003). Wolframin is a transmembrane glycoprotein consisting of 890 amino acids with a molecular mass of 100.29 kD (Takeda et al., 2001). It is composed of nine transmembrane segments and is embedded in the membrane with an N(cyt)/C(lum) topology (Hofmann et al., 2003). Its function has not yet been fully clarified. Some studies (Fonseca et al., 2010; Hara et al., 2014) have shown that *WFS1* appears to be important to the negative regulation of ER stress. Osman et al. (2003) proposed that wolframin may play an important role in the regulation of intracellular Ca^2+^ homeostasis. In addition, Zatyka et al. (2008) noted that an interaction with *WFS1* is essential for Na^+^/K^+^ ATPase beta-1 subunit maturation in the ER and that the loss of this interaction may contribute to the pathogenesis of WS1.

The Human Gene Mutation Database (HGMD) has so far recorded 393 variants in *WFS1*. Except for WS1, it is also thought to underlie some autosomal dominant conditions that are less severe. Given the clinical and genetic heterogeneity of WS1, combining genetic diagnosis with clinical features is an effective supplementary diagnostic method. In addition, genetic diagnosis is essential for prognosis evaluation, individualized treatment, genetic counseling and carrier screening. At present, limited data are available on which *WFS1* mutations are associated with WS1 in the Chinese population, and only a small number of case reports have been published. No prevalence has been reported in China. In this study, we used NGS to evaluate five Chinese patients affected by WS1, thus increasing the spectrum of *WFS1* mutations by adding four novel variants. We have also reviewed their clinical features.

## Materials and Methods

### Subjects and Ethics Statement

We collected 279 patients with unexplained OA who presented at the Eye and ENT Hospital of Fudan University from 2016 to 2018. Patients with infection, ischemia, optic neuritis, myelitis, and tumors were excluded. The study was performed according to the Declaration of Helsinki and approved by the Ethics Committee of the Eye and ENT Hospital of Fudan University. Written informed consent was obtained from all the subjects or their guardians to participate in this study and for the publication of these data.

### Clinical Evaluations

All patients underwent detailed clinical evaluations, including medical history, family history, and basic and auxiliary examinations. The chief complaint, the course of the disease and accompanying symptoms were recorded in detail. In addition, every patient was asked whether his parents were consanguineous and whether other family members were affected. Basic and auxiliary examinations included best corrected visual acuity, tonometry, color vision (Ishihara color plate), slit-lamp examination, dilated fundus examination, frequency domain optical coherence tomography (Cirrus OCT 5000, Carl Zeiss Meditec, Inc., Dublin, CA, United States), Humphrey VisualField Analyzer (Carl Zeiss Meditec, Inc., Dublin, CA, United States), full-field electroretinography (ERG), and visual evoked potential (VEP) (LKC UTAS E3000 LKC Technologies, Inc., United States) tests. A preliminary assessment of systemic conditions was performed by measuring the fasting blood glucose concentration, pure-tone audiometry, and urine specific gravity. Orbital magnetic resonance imaging or computed tomography was used to exclude neoplasms.

### Genetic Analyses

We designed a high-throughput microarray in collaboration with BGI-Shenzhen (Shenzhen, Guangdong, China). It contained the exon sequences of 792 genes (**Supplementary Table S1**) as well as 30 bp on either side of the exonic region, selected on the basis of OMIM database, the ophthalmic disease gene database, and related literature. Only genes known to be involved in human inherited eye diseases, including syndromes with ocular manifestation, were selected. Genes found only in animal models of eye diseases were not included. Genomic DNA was extracted from peripheral blood obtained from the probands and their family members using FlexiGene DNA Kits (Qiagen, Venlo, the Netherlands) according to standard procedures, sheared into 150–250 bp fragments, and captured with the Agilent SureSelect Target Enrichment Kit (Agilent Technologies, Inc., USA). Enriched libraries were sequenced on the Illumina HiSeq 2000 platform (Illumina, Inc., San Diego, CA, United States), and reads were aligned to the reference human genome (UCSC hg38) using the Burrows–Wheeler aligner version 0.7.10 (BWA-MEM). The following databases were used for the annotation: 1,000 Genomes Project, 1,000 Genomes in the East Asian population, Single Nucleotide Polymorphism Database, the Exome Aggregation Consortium, and Genome Aggregation Database. Then, we retained variants with a minor allele frequency (MAF) < 0.1% to filter out possible non-deleterious variants based on MAF of each variant in these five databases. In addition, we performed the variant prioritizations based on MAF, potential deleterious effect and mutation reports in public databases such as HGMD, OMIM, and ClinVar. Furthermore, we analyzed the evolutionary conservation of the mutations online. Finally, Sanger sequencing and cosegregation analyses were performed within families. If no suspected pathogenic variant was found, Sanger sequencing was performed to check for the presence of the three primary mitochondrial DNA mutations (m.3460G > A, m.11778G > A, m.14484T > C) (Yen et al., 2006). The cDNA NM_006005.3 and protein NP_005996.2 sequences were used for the mutation nomenclature. Variants were classified according to the American College of Medical Genetics.

## Results

**Figure 1:**
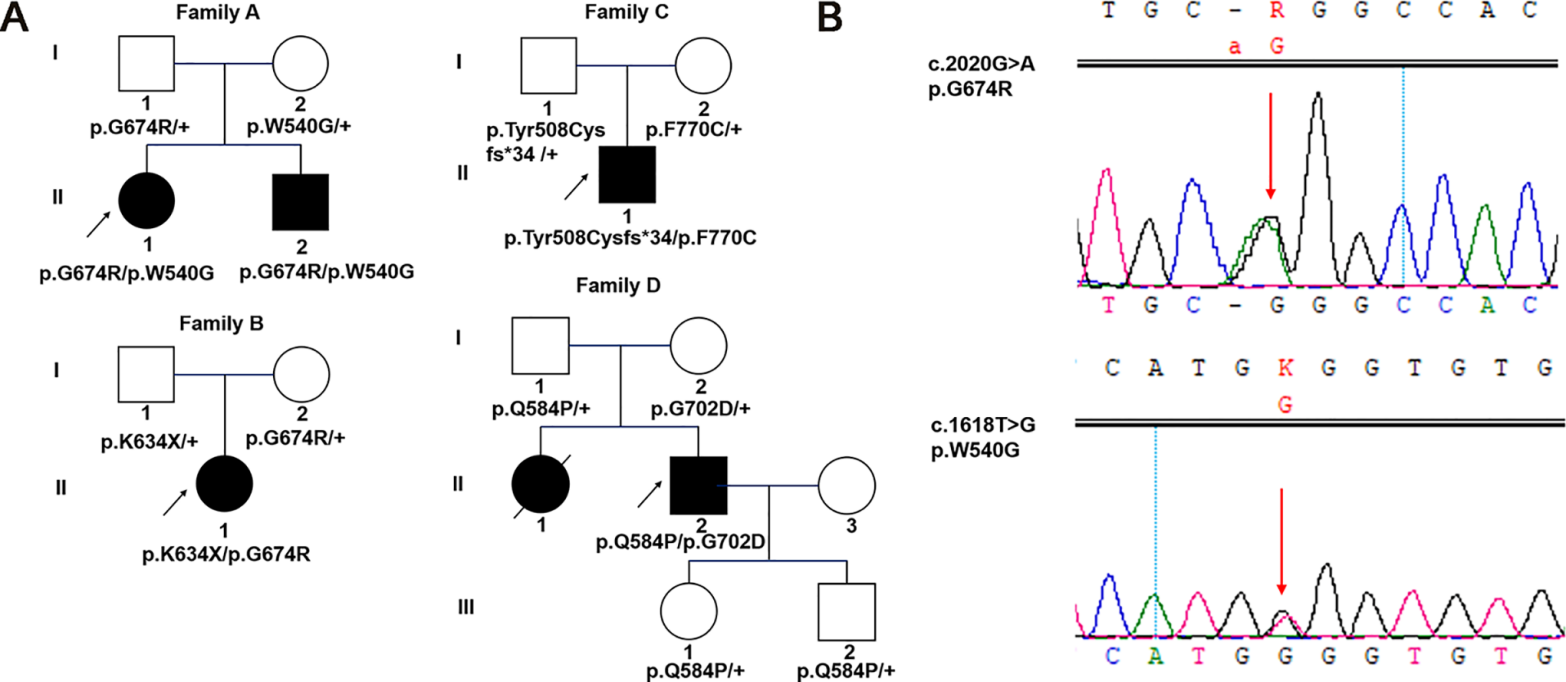
Pedigrees and identified variants in five Chinese patients with WS1. **(A)** Squares represent males, circles represent females, empty symbols represent normal controls, filled symbols represent affected patients, and arrows indicate the proband. **(B)** Sanger sequencing results for *WFS1* mutations in family A-1. Arrows indicate the positions of the variants.

**Figure 2:**
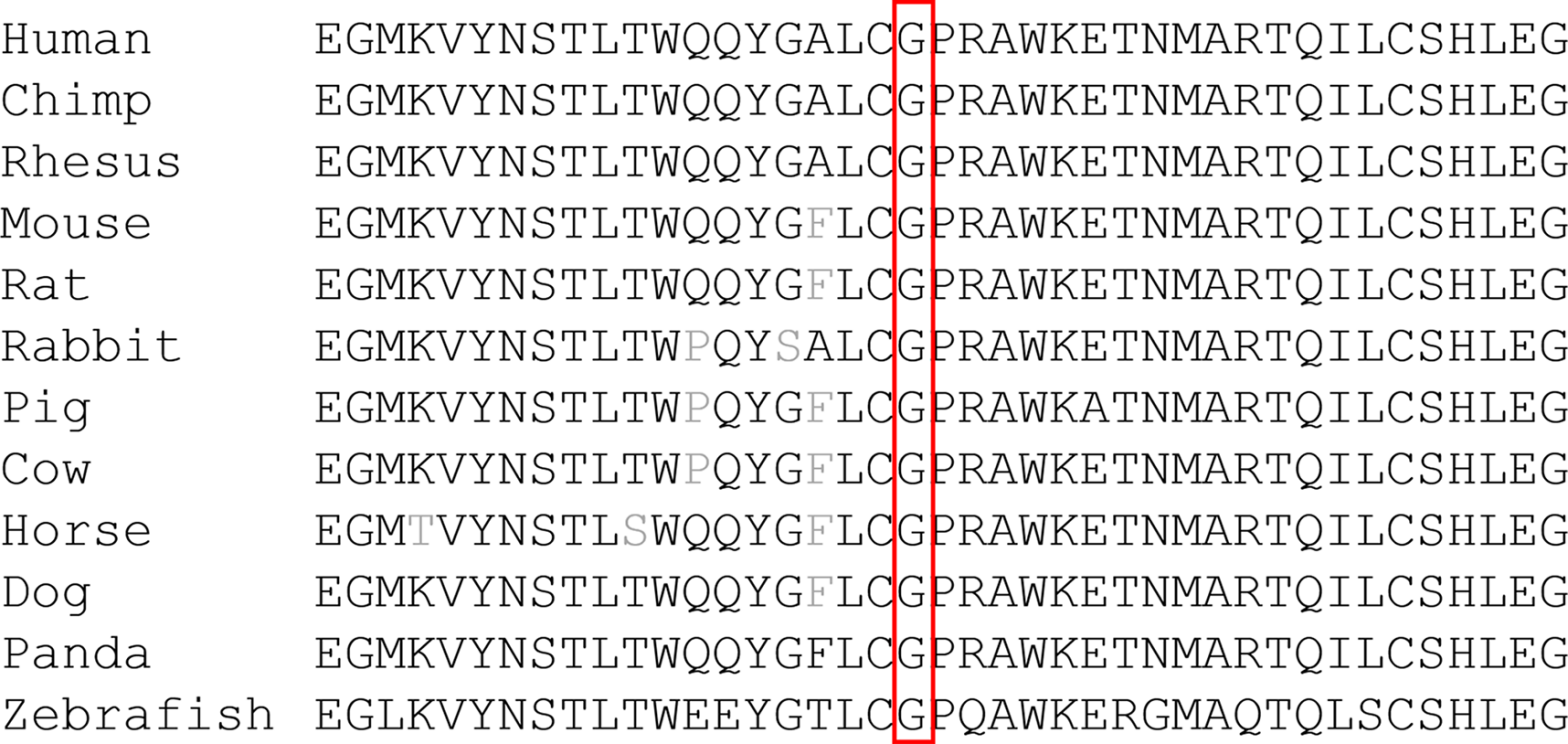
Protein sequence alignment of human *WFS1* and its orthologs. Codon 674 and its subsequent sequences were highly conserved amino acids in *WFS1* across different species.

Of the 279 patients with unexplained OA, five from four unrelated families carried compound heterozygous mutations in *WFS1*, and all the variants cosegregated with the phenotypes observed in these families. The main clinical and genetic characteristics of the five patients are listed in **Table 1** and **Table 2**, and the pedigrees of the four families (A–D) are presented in **Figure 1A**. Family history was clearly reported in three patients, whereas two patients were sporadic. Seven variants of *WFS1* were identified; these included four novel variants (p.W540G, p.K634*, p.F770C, and p.Q584P) and three known variants (P.G674R, P.Tyr508Cysfs*34, and P.G702D) (Khanim et al., 2001; Aluclu et al., 2006; Gasparin et al., 2009). To date, 47 Chinese cases with WS1 have been published in pubMed (Https://Www.Ncbi.Nlm.Nih.Gov/Pubmed/), wanfang data (Http://G.Wanfangdata.Com.Cn/Index.Html), and China National Knowledge Infrastructure (Http://Www.Cnki.Net/) (Sun 1986; Nan and Cou, 1987; Wu 1996; Long et al., 1997; Shi et al., 1998; Feng and Chen, 2002; Chai et al., 2003; Li et al., 2003; Wang and Wang, 2004; Fang et al., 2005; Li et al., 2005; Shi et al., 2005; Lv et al., 2006; Lu et al., 2008; Zhu et al., 2008; Hong et al., 2009; Zhang et al., 2009; Xu and Li, 2011; Fan et al., 2013; Wang and Li, 2011; Xu et al., 2013; Peng et al., 2015; Wang et al., 2014; Du et al., 2017; Ni et al., 2017; Duan et al., 2018). However, only 10 patients were confirmed by genetic testing (**Table 3**), and only nine variants of *WFS1* have been reported in the Chinese population. Our study reports five Chinese patients and further enriches the spectrum of *WFS1* mutations. In addition, It is worth noting that c.2020G > A(P.G674R) was recurrent in this study and was identified in two unrelated families. The allele frequency of this variant in East Asian populations is 0.000231965 according to the gnomAD database. Multiple orthologous sequence alignment (MSA) around codon 674 demonstrated this and neighboring residues are highly conserved amino acids in *WFS1* (**Figure 2**). Furthermore, four prediction tools, including mutationtaster software, SIFT, LRT, and FATHMM, predicted that this variant is deleterious. Therefore, it suggested that p.G674R is likely to be a pathogenic variant with higher prevalence in the Chinese population.

**Table 1:** Clinical characteristics and onset age of patients with WS1 in this study.

Patient	Sex	Age	DM	OA	DI	HD	UD	ND	Other
A-1	M	26	11	7	–	26	–	–	–
A-2	F	27	12	17	–	–	Nephritis	–	–
B	F	12	–	9	–	–	–	–	–
C	M	37	–	11	–	–	–	–	–
D	M	28	8	8	–	–	28	28	Hypogonadism

M, male; F, female; DM, diabetes mellitus; OA, optic atrophy; DI, diabetes insipidus; HD, hearing defects; UD, urological or renal defects; ND, neurological defects; WS1, Wolfram syndrome type 1.

**Table 2:** Mutations in *WFS1* found in 5 Chinese patients.

Family	Exon	Nucleotide change	Amino acid change	Type of mutation	References
A-1, 2	8	c.1618T > G	p.W540G	Missense	This study
	8	c.2020G > A	p.G674R	Missense	Khanim et al., 2001
B	8	c.1900A > T	p.K634*	Nonsense	This study
	8	c.2020G > A	p.G674R	Missense	Khanim et al., 2001
C	8	c.1522_1523delTA	p.Tyr508Cysfs*34	Frameshift	Aluclu et al., 2006
	8	c.2309T > G	p.F770C	Missense	This study
D	8	c.1751A > C	p.Q584P	Missense	This study
	8	c.2105G > A	p.G702D	Missense	Gasparin et al., 2009

All patients carried two compound heterozygous mutations in the WFS1 gene.

**Table 3:** *WFS1* mutations previously identified in Chinese patients with WS1.

Family	Exon	Nucleotide change	Amino acid change	Allele status	Type of mutation	References
E	8	c.1760G > A	p.R587Q	Hom	Missense	Duan et al., 2018
F	8	c.1250_1252delTCT	p.Phe417del	Hom	Deletion	Fang et al., 2005
G	8	c.2411T > C	p.L804P	Hom	Missense	Xu et al., 2013
H	8	c.1300_1302delGTC	p.Val434del	Het	Deletion	Hong et al., 2009
	8	c.1997G > A	p.W666*	Het	Nonsense	Hong et al., 2009
I	8	c.1300_1302delGTC	p.Val434del	Het	Deletion	Duan et al., 2018
	4	c.453_460delCAGAAGAG	p.Asp151Glufs*93	Het	Frameshift	Duan et al., 2018
J	8	c.1600T > G	p.Y534D	Hom	Missense	Li et al., 2005
K	8	c. 2196_2218del23bp	p.Cys733fs*749	Hom	Frameshift	Peng et al., 2015
L	4	c.433G > A	p.C148Y	Hom	Missense	Ni et al., 2017

Het, heterozygous; Hom, homozygous; WS1, Wolfram syndrome type 1. The other two patients were identified as having three WFS1 mutations in exon 8, but detailed results were unavailable (Duan et al., 2018).

### Family A

A 27-year-old female patient came for genetic testing. She was found to have DM when she was 12 years old and was diagnosed with bilateral OA 5 years later. In addition, her medical history revealed nephritis. She had an affected younger brother with similar symptoms. He presented with OA when he was 7 years old and was diagnosed with DM at the age of 11. Pure-tone audiometry revealed sensorineural hearing loss, while his urine-specific gravity was normal. Their vision was 20/400 OU. Genetic analysis identified a reported variant c.2020G > A (p.G674R, inherited paternally) (Khanim et al., 2001) and a novel variant c.1618T > G (p.W540G, inherited maternally) of *WFS1* in both the proband and her brother (**Figure 1B**). Parental examination and testing were normal.

### Family B

A 12-year-old girl was found to have bilateral OA when she was 8 years old. She was the only affected family member. Past medical history showed a normal delivery, normal growth and development. Best corrected visual acuity was 20/30 with -4.50 sph OD and -0.25 sph OS, and color vision was abnormal. Frequency domain optical coherence tomography showed that the retinal nerve fiber layer (RNFL) and macular ganglion cell complex were thinning in four quadrants. The visual field revealed local decreased visual sensitivity in the right eye and a central scotoma in the left eye. Fundus examination showed that the temporal optic nerve was pale in the right eye and that all quadrants were pale in the left eye, while full-field ERG and VEP were normal. Orbital magnetic resonance imaging confirmed bilateral OA. In addition, pure-tone audiometry, urine specific gravity and fasting blood glucose concentration were normal. Genetic analysis showed a previously reported pathogenic variant associated with WS1 in her *WFS1* gene (c.2020G > A, p.G674R) (Khanim et al., 2001), which was inherited from her clinically normal mother. In addition, there was a novel variant c.1900A > T (p.K634*) identified in the girl, which was inherited from her clinically normal father.

### Family C

A 37-year-old male patient came for evaluation of OA, with which he was diagnosed when he was 11 years old. Examination revealed visual acuity of 20/400 in each eye. Fundus examination showed pale optic discs in both eyes. Pattern VEP showed that the time to the P1 wave peak was delayed and its amplitude diminished. In addition, visual fields were constricted in both eyes. The peripapillary RNFL was thinning in the superior and inferior quadrants while macular ganglion cell complex was thinning in all four quadrants. Orbital computed tomography confirmed bilateral OA. He had no systemic findings. Pure-tone audiometry, urine specific gravity and fasting blood glucose concentration were normal. Genetic analysis found a reported variant c.1522_1523delTA (p.Tyr508Cysfs*34, inherited paternally) (Aluclu et al., 2006) and a novel variant c.2309T > G (p.F770C, inherited maternally) of *WFS1* in the proband. His mother was diagnosed with DM 10 years prior. Parental ophthalmic examination and testing were normal.

### Family D

A 28-year-old male patient presented for genetic counseling. He presented with bilateral OA when he was 8 years old and was diagnosed with DM 20 years prior along with the clinical manifestations of polydipsia and polyuria. He presented with polyphagia and transient hand tremor for 3 months. In addition, he had enuresis, urgent and frequent urination, impotence, premature ejaculation and epididymitis. He had been constipated in recent months. Examination revealed visual acuity of 20/200 and 20/300 in the right and left eyes, respectively. The peripapillary RNFL showed thinning in the superior, temporal and inferior quadrants of the left eye, while thinning was found in all four quadrants of the right eye. VEP and ERG were abnormal, and he had visual field defects. Family history revealed that he had an affected sister who had poor vision since childhood and died of nephritis at the age of 25. Parental examination and testing were normal. Genetic analysis showed that he carried compound heterozygous mutations of *WFS1*: a reported variant c.2105G > A (p.G702D, inherited maternally) (Gasparin et al., 2009) and a novel variant c.1751A > C (p.Q584P, inherited paternally). His clinically normal son and daughter both carried a heterozygous variant c.1751A > C (p.Q584P) of *WFS1*.

## Discussion

In this study, by using genetic testing based on NGS we were able to diagnose five WS1 patients out of 279 patients with previously unexplained OA. The clinical diagnostic criteria for WS1 are juvenile-onset DM and OA. It is very difficult to diagnose WS1 at an early stage, and this is especially true for atypical WS1. NGS is a high-throughput approach for efficiently sequencing large gene pools at lower costs and can be used to identify rare disease genes. In families B and C, while the two probands presented with only OA, genetic analysis based on NGS made it possible to diagnose and confirm WS1 early. WS1 is a progressive neurodegenerative disease mainly affecting pancreatic beta cells and neuronal cells (Gerbitz, 1999; Yamada et al., 2006). Therefore, early diagnosis is crucial for patients and their families. This is beneficial to the systematic evaluation, monitoring and management of complications to improve the quality of life and delay the progression of the disease. Finally, it provides a genetic basis for carrier testing, and if desired, for preimplantation diagnostic testing.

Although the HGMD has recorded 263 *WFS1* mutations associated with WS1 thus far, 62.5% (5/8) of the mutations identified in our study were novel. Among the 263 identified mutations, there were 109 missense mutations, 50 nonsense mutations, 60 small deletion mutations, and 25 small insertion mutations. These mutations were mainly concentrated in exon 8. Khanim F et al.(Khanim et al., 2001) identified the mutations in *WFS1* in 90% of WS1 patients and found they were mainly compound heterozygous mutations, in accordance with our study. In family A, the proband and her brother had the same mutations in *WFS1*, but their phenotypes were not exactly the same. D'Annunzio et al. (2008) evaluated six WS1 patients in five Italian families and noted that the same mutation could lead to different phenotypes, also consistent with our study. This finding suggested that the pathogenesis of WS1 was not only dependent on the mutation of *WFS1*, but also may be related to other genetic or environmental factors, such as modifier genes.

de Heredia et al. (2013) analyzed genetic data in 412 WS1 patients reported since 1998, and found no major mutation hot spots; only the following six mutations were found in more than 5% of patients: c.2649delC (p.Phe884Serfs*68, 7.42%), c.1230_1233del (p.Val412Serfs*29, 6.82%), c.409_424dup (p.Val142Glyfs*110, 6.53%), c.2119G > A (p.V707I, 6.23%), c.1362_1377del (p.Tyr454*, 5.64%), and c.1243_1245del (p.Val415del, 5.34%). In our study, c.2020G > A (p.G674R) was the most prevalent alteration in our Chinese patients and was highly conserved in different species during evolution. This suggests that the mutation may impair the key functions of the protein. Several studies have reported that the variant c.2020G > A (p.G674R) combined with c.2020G > A (p.G674R) and c.1620delGTG (Trp540del) respectively resulted in the occurrence of WS1 (Khanim et al., 2001; Aloi et al., 2012). However, more samples are required to determine whether this is a common pathogenic variant in the Chinese population.

At present, only 47 Chinese WS1 patients have been reported since 1986. Furthermore, only 10 of these patients were confirmed by genetic testing. Given the specificity of ethnicity and the complexity of genotype-phenotype relationship, large cohort studies in the future are essential to elucidate the genotype–phenotype relationship of WS1 in the Chinese population. Our study further enriches the spectrum of *WFS1* mutations, which is currently based on limited data, making our findings of great significance, especially in the Chinese population.

## Conclusion

NGS has become a powerful method for the detection of ophthalmic disorders with high clinical and genetic heterogeneity. Implementing comprehensive measures to slow the progression of diseases is essential not only in early diagnosis but also in risk evaluation, individualized treatment and carrier screening for probands and their families. Precise diagnosis based on clinical manifestation and genetic testing will become the gold standard for the diagnosis of hereditary eye diseases and syndromes in the future.

## Ethics Statement

The study was performed according to the Declaration of Helsinki and approved by the Ethics Committee of the Eye and ENT Hospital of Fudan University. Written informed consent was obtained from all the subjects or their guardians to participate in this study and for the publication of these data.

## Author Contributions

J-HW conceived and designed this study. G-HT and J-HW recruited patients, performed clinical examinations and interpretation. F-JG, F-YH, PX, S-HZ, and D-DW collected the clinical samples and clinical data. F-JG and D-DW analyzed the sequencing data. J-HW and D-DW wrote and revised the manuscript.

## Funding

This work was supported by grants from the National Natural Science Foundation of China (no. 81770925 and no. 81790641).

## Conflict of Interest

The authors declare that the research was conducted in the absence of any commercial or financial relationships that could be construed as a potential conflict of interest.

## Supplementary Material

Supplementary Table 1 792 genes in panel based on NGS.

## References

[B1] Aloi C. Salina A. Pasquali L. Lugani F. Perri K. Russo C. (2012). Wolfram syndrome: new mutations, different phenotype. PLoS ONE 7, e29150. 10.1371/journal.pone.0029150 22238590 PMC3251553

[B2] Aluclu M. U. Bahceci M. Tuzcu A. Arikan S. Gokalp D. (2006). A new mutation in WFS1 gene (C.1522-1523delTA, Y508fsX421) may be responsible for early appearance of clinical features of wolfram syndrome and suicidal behaviour. Neuro Endocrinol. Lett 27, 691–694. 17187023

[B3] Barrett T. G. Bundey S. E. Macleod A. F. (1995). Neurodegeneration and diabetes: UK nationwide study of Wolfram (DIDMOAD) syndrome. Lancet 346, 1458–1463. 10.1016/S0140-6736(95)92473-6 7490992

[B4] Chai H. Q. Lu Y. Q. Sun W. H. Xie Q. L. Lu G. Y. Guo L. (2003). A case of Wolfram syndrome with anosmia and neurogenic bladder. Jiangsu Med. J. 29, 291. 10.19460/j.cnki.0253-3685

[B5] D'Annunzio G. Minuto N. D'Amato E. de Toni T. Lombardo F. Pasquali L. (2008). Wolfram syndrome (diabetes insipidus, diabetes, optic atrophy, and deafness): clinical and genetic study. Diabetes Care 31, 1743–1745. 10.2337/dc08-0178 18566338 PMC2518337

[B6] de Heredia M. L. Cleries R. Nunes V. (2013). Genotypic classification of patients with Wolfram syndrome: insights into the natural history of the disease and correlation with phenotype. Genet. Med. 15, 497–506. 10.1038/gim.2012.180 23429432

[B7] Du W. Li J. Y. Yin R. Liu Y. Q. Wang T. Liu H. (2017). A case of incomplete Wolfram syndrome with stroke in youth. Chin. J. Clin. Neurosci. 25, 559–563.

[B8] Duan L. Li Q. Tong A. L. Mao J. F. Yu M. Yuan T. (2018). Clinical characteristics of wolfram syndrome in chinese population and a novel frameshift mutation in WFS1 . Front. Endocrinol. (Lausanne) 9, 18. 10.3389/fendo.2018.00018 29483894 PMC5816339

[B9] Fan Y. S. Luo J. H. Yu R. P. Liu B. (2013). A case of Wolfram syndrome with ventricular septal defect. Chin. J. Prac. Intern. Med. 33, 331–332.

[B10] Fang Q. C. Jia W. P. Zhang R. Li Q. Hu C. Shao X. Y. (2005). novel mutation of WFS1 gene in Chinese patients with Wolfram syndrome. Natl. Med. J. China 85, 2468–2471. 10.3760/j:issn:0376-2491.2005.35.007 16321270

[B11] Feng D. S. Chen X. (2002). Wolfram syndrome with visual and hearing impairment: a case report. Chin. J. Coal Ind. Med. 5, 1233.

[B12] Fonseca S. G. Ishigaki S. Oslowski C. M. Lu S. Lipson K. L. Ghosh R. (2010). Wolfram syndrome 1 gene negatively regulates ER stress signaling in rodent and human cells. J. Clin. Invest. 120, 744–755. 10.1172/JCI39678 20160352 PMC2827948

[B13] Fraser F. C. Gunn T. (1977). Diabetes mellitus, diabetes insipidus, and optic atrophy. An autosomal recessive syndrome? J. Med. Genet. 14, 190–193. 10.1136/jmg.14.3.190 881709 PMC1013555

[B14] Gasparin M. R. Crispim F. Paula S. L. Freire M. B. Dalbosco I. S. Manna T. D. (2009). Identification of novel mutations of the WFS1 gene in Brazilian patients with Wolfram syndrome. Eur. J. Endocrinol. 160, 309–316. 10.1530/EJE-08-0698 19042979

[B15] Gerbitz K. D. (1999). Reflexions on a newly discovered diabetogenic gene, wolframin (WFS1). Diabetologia 42, 627–630. 10.1007/s001250051205 10333058

[B16] Hara T. Mahadevan J. Kanekura K. Hara M. Lu S. Urano F. (2014). Calcium efflux from the endoplasmic reticulum leads to beta-cell death. Endocrinol. 155, 758–768. 10.1210/en.2013-1519 PMC3929724 24424032

[B17] Hofmann S. Philbrook C. Gerbitz K. D. Bauer M. F. (2003). Wolfram syndrome: structural and functional analyses of mutant and wild-type wolframin, the WFS1 gene product. Hum. Mol. Genet. 12, 2003–2012. 10.1093/hmg/ddg214 12913071

[B18] Hong J. Zhang Y. W. Zhang H. J. Jia H. Y. Zhang Y. Ding X. Y. (2009). The novel compound heterozygous mutations, V434del and W666X, in WFS1 gene causing the Wolfram syndrome in a Chinese family. Endocrine 35, 151–157. 10.1007/s12020-009-9145-7 19160074

[B19] Inoue H. Tanizawa Y. Wasson J. Behn P. Kalidas K. Bernal-Mizrachi E. (1998). A gene encoding a transmembrane protein is mutated in patients with diabetes mellitus and optic atrophy (Wolfram syndrome). Nat. Genet. 20, 143–148. 10.1038/2441 9771706

[B20] Khanim F. Kirk J. Latif F. Barrett T. G. (2001). WFS1/wolframin mutations, Wolfram syndrome, and associated diseases. Hum. Mutat. 17, 357–367. 10.1002/humu.1110 11317350

[B21] Li D. L. Zhu J. Zhang X. M. (2003). Wolfram syndrome: one case report. Chin. J. Endocrinol. Metab. 19, 329.

[B22] Li Q. Jia W. P. Fang Q. C. (2005). Two cases of Wolfram syndrome. Zhonghua Nei Ke Za Zhi 44, 860–861.

[B23] Long X. R. Li Q. Y. Wang Z. C. Li C. J (1997). Three cases of wolfram syndrome. Chin. J. Pediatr. 38, 430.

[B24] Lu W. Li Q. Jia W. P. (2008). Nursing care of one patient with Wolfram syndrome. Nurs. Res. Chin. 22, 463–464.

[B25] Lv L. Ye J. Liu J. Y. Liu Y. J. Huang L. (2006). Wolfram syndrome: a case report. Chin. J. Appl. Clin. Pediatr. 21, 591.

[B26] Matsunaga K. Tanabe K. Inoue H. Okuya S. Ohta Y. Akiyama M. (2014). Wolfram syndrome in the Japanese population; molecular analysis of WFS1 gene and characterization of clinical features. PLoS ONE 9, e106906. 10.1371/journal.pone.0106906 25211237 PMC4161373

[B27] Medlej R. Wasson J. Baz P. Azar S. Salti I. Loiselet J. (2004). Diabetes mellitus and optic atrophy: a study of Wolfram syndrome in the Lebanese population. J Clin Endocrinol. Metab 89, 1656–1661. 10.1210/jc.2002-030015 15070927

[B28] Nan G. Z. Cou C. (1987). A case of Wolfram syndrome. Chin. J. Endocrinol. Metab. 46, 234.

[B29] Ni J. W. Sun C. J. Cheng R. Q. Zhao Z. H. Li X. J. Pei Z. (2017). A case of Wolfram syndrome caused by a new mutation of WFS1 gene. Chin. J. Pediatr. 55, 68–69.

[B30] Osman A. A. Saito M. Makepeace C. Permutt M. A. Schlesinger P. Mueckler M. (2003). Wolframin expression induces novel ion channel activity in endoplasmic reticulum membranes and increases intracellular calcium. J. Biol. Chem. 278, 52755–52762. 10.1074/jbc.M310331200 14527944

[B31] Peng J. X. Wang Z. M. Jiang L. M. He Y. F. Hu Z. W. Jia X. L. (2015). Clinical and genetic analysis of WFS1 gene in a case of Wolfram syndrome. Chin. J. Endocrinol. Metab. 31, 731–734. 10.3760/cma.j.issn.1000-6699.2015.08.017

[B32] Shi C. H. Song X. L. Sun J. (1998). Wolfram syndrome in mother and daughter: two cases report. Chin. J. Endocrinol. Metab. 14, 92.

[B33] Shi X. Y. Liu Y. L. Wang L. (2005). A case of Wolfram syndrome. Chin. J. Endocrinol. Metab. 21, 384.

[B34] Strom T. M. Hortnagel K. Hofmann S. Gekeler F. Scharfe C. Rabl W. (1998). Diabetes insipidus, diabetes mellitus, optic atrophy and deafness (DIDMOAD) caused by mutations in a novel gene (wolframin) coding for a predicted transmembrane protein. Hum. Mol. Genet. 7, 2021–2028. 10.1093/hmg/7.13.2021 9817917

[B35] Sun R. Y. (1986). Wolfram syndrome: a case report. Beijing Med. J. 4, 221. 10.15932/j.0253-9713

[B36] Takeda K. Inoue H. Tanizawa Y. Matsuzaki Y. Oba J. Watanabe Y. (2001). WFS1 (Wolfram syndrome 1) gene product: predominant subcellular localization to endoplasmic reticulum in cultured cells and neuronal expression in rat brain. Hum. Mol. Genet. 10, 477–484. 10.1093/hmg/10.5.477 11181571

[B37] Wang Y. Y. Li F. P. (2011). Two cases of Wolfram syndrome. Lingnan J. Emerg. Med. 18, 58–59. 10.3969/j.issn.1671-301X.2013.01.026

[B38] Wang L. P. Wang X. L. (2004). A case report of special Wolfram syndrome. J. Chin. Neurol. 17, 220.

[B39] Wang X. J. Zhai W. J. Liu Q. Zheng D. P. (2014). Nursing care of patients with Wolfram syndrome. J. Nursing Sci. 29, 40–41. 10.3870/hlxzz.2014.17.040

[B40] Wolfram D. J. Wagener H. P. (1938). Diabetes mellitus and simple optic atrophy among siblings: report of four cases. Mayo. Clin. Proc. 13, 715–718.

[B41] Wu Y. Y. (1996). Wolfram syndrome: a case report. Chin. J. Diabetes 4, 176.

[B42] Xu Q. Qu H. Wei S. (2013). Clinical and molecular genetic analysis of a new mutation in children with Wolfram syndrome: a case report. Mol. Med. Rep. 7, 965–968. 10.3892/mmr.2013.1277 23338790

[B43] Xu L. Y. Li B. (2011). A case of incomplete Wolfram syndrome. Chin. J. Pract. Pediatr. 26, 240.

[B44] Yamada T. Ishihara H. Tamura A. Takahashi R. Yamaguchi S. Takei D. (2006). WFS1-deficiency increases endoplasmic reticulum stress, impairs cell cycle progression and triggers the apoptotic pathway specifically in pancreatic beta-cells. Hum. Mol. Genet. 15, 1600–1609. 10.1093/hmg/ddl081 16571599

[B45] Yamamoto H. Hofmann S. Hamasaki D. I. Yamamoto H. Kreczmanski P. Schmitz C. (2006). Wolfram syndrome 1 (WFS1) protein expression in retinal ganglion cells and optic nerve glia of the cynomolgus monkey. Exp. Eye Res. 83, 1303–1306. 10.1016/j.exer.2006.06.010 16928372

[B46] Yen M. Y. Wang A. G. Wei Y. H. (2006). Leber's hereditary optic neuropathy: a multifactorial disease. Prog. Retin. Eye Res. 25, 381–396. 10.1016/j.preteyeres.2006.05.002 16829155

[B47] Zatyka M. Ricketts C. Da S. X. G. Minton J. Fenton S. Hofmann-Thiel S. (2008). Sodium-potassium ATPase 1 subunit is a molecular partner of Wolframin, an endoplasmic reticulum protein involved in ER stress. Hum. Mol. Genet. 17, 190–200. 10.1093/hmg/ddm296 17947299 PMC6101208

[B48] Zhang Y. W. Hong J. Zhang H. J. Jia H. Y. Cui B. Wang W. Q. (2009). Two novel mutations of WFS1 gene in a Chinese family with Wolfram syndrome. Shanghai Med. J. 32, 431–433.

[B49] Zhu X. Y. Dong Z. X. Su Z. F. Li J. Huang H. Y. (2008). A case of Wolfram syndrome with ataxia. Chin. J. Neurol. 41, 870–871.

